# Multiple organ dysfunction syndrome caused by fish gallbladder poisoning: A case report of delayed etiological diagnosis

**DOI:** 10.1097/MD.0000000000042322

**Published:** 2025-05-09

**Authors:** Xiang Chen, Jing-Ping Cheng, Ling-Ling Xu, Jia-Xin Chen, Ying-Chun Zhang

**Affiliations:** a Department of Gerontology, CR & WISCO General Hospital Affiliated to Wuhan University of Science and Technology, Wuhan, Hubei Province, China; b Medical College, School of Medicine, Wuhan University of Science and Technology, Wuhan, Hubei Province, China.

**Keywords:** case report, delayed diagnosis of the cause, fish gallbladder poisoning, multiple organ dysfunction syndrome, murtagh safe diagnosis and treatment

## Abstract

**Rationale:**

Fish gallbladder poisoning is an acute poisoning caused by eating fish gallbladders, which can damage the functions of multiple organs such as the heart, liver, and kidneys, and can be fatal in severe cases.

**Patient concerns:**

A 56-year-old female farmer, believing in a folk remedy, consumed the gallbladder of a black carp (*Mylopharyngodon piceus*) weighing approximately 4 kg to treat her cough. She subsequently developed abdominal pain and diarrhea for 6 days, followed by 1 day of anuria.

**Diagnoses:**

Fish gallbladder poisoning and multiple organ dysfunction syndrome.

**Interventions:**

The patient underwent oxygen therapy, anti-infective treatment, hepatoprotective measures, acid suppression and gastric protection, anti-heart failure management, correction of anemia, maintenance hemodialysis, and symptomatic and supportive care.

**Outcomes:**

After nearly 2 weeks of hospitalization, the patient’s condition improved. Following discharge, she continued maintenance hemodialysis for approximately 3 months, until her renal function was restored.

**Lessons:**

Fish gallbladder poisoning is primarily caused by toxins such as histamine, hydrocyanic acid, and cyprinol sulfate, leading to severe multiorgan dysfunction, particularly acute kidney injury. Early recognition, prompt hemodialysis, and comprehensive supportive treatment are essential for a favorable prognosis. Maintaining clinical vigilance and providing public health education on the dangers of consuming fish gallbladders are crucial for preventing similar incidents.

## 1. Introduction

In the past few decades, fish gallbladder poisoning has been reported worldwide and is gradually increasing. According to incomplete statistics, 451 people were poisoned by swallowing toxic fish gallbladders in China between 1964 and 1999, of whom 83 died, with a mortality rate of 18.04%.^[1]^ The incidence of digestive system damage (digestive tract and liver disease) and urinary system damage (nephritis and renal failure) in patients with acute fish gallbladder poisoning is 100%, accompanied by electrolyte disorders, circulatory system damage, and nervous system damage.^[2]^ Fish gallbladder poisoning typically involves pathogenic chemical toxins such as bile acids, histamine, and hydrocyanic acid. These substances exert direct toxic effects on multiple organs and can be fatal in severe cases. Risk factors include the mistaken belief that fish gallbladders have therapeutic value for ailments such as sore throat, asthma, and eye diseases, leading to their consumption. From a geographical perspective, fish gallbladder poisoning is most frequently observed in rural areas or among populations with limited medical knowledge, where folk remedies remain popular. Therefore, fish gallbladder poisoning is a serious health problem that should be taken seriously and vigilantly. Here, we report a case of multiple organ dysfunction syndrome (MODS) caused by fish gallbladder poisoning due to delayed etiology diagnosis caused by clinical thinking anchoring and cognitive bias. This case underscores the importance of comprehensive history-taking and maintaining a broad differential diagnosis.

## 2. Case report

A 56-year-old female farmer was admitted to our intensive care unit with a 6-day history of abdominal pain and diarrhea, followed by 1 day of anuria. Six days earlier, she had received supportive treatment at another hospital, which significantly alleviated her abdominal pain and diarrhea; however, anuria developed 1 day before admission. Her past medical history was unremarkable, except for a cough 2 weeks prior to admission, during which she took a commonly used traditional Chinese medicine for cough relief on 1 occasion. Upon admission, laboratory results indicated: white blood cell count of 19.81 × 10^9^/L, hemoglobin of 94 g/L, platelet count of 103 × 10^9^/L, alanine aminotransferase (ALT) of 888.6 U/L, aspartate aminotransferase (AST) of 131.4 U/L, urea of 34.04 mmol/L, creatinine of 837.8 μmol/L, B-type natriuretic peptide of 5301 pg/mL, and myoglobin of 1023.1 ng/mL, as shown in Tables 1 and 2. Ultrasound revealed a small amount of pericardial effusion. Chest and abdominal CT scans indicated bilateral pulmonary infection, pleural effusion, ascites, possible cholecystitis, pelvic effusion, and abdominal wall edema. No other significant abnormalities were detected on additional examinations.

**Table 1:** Dynamic changes of blood routine in our hospital.

	Hospitalization day 2	Hospitalization day 3	Hospitalization day 4	Hospitalization day 5	Hospitalization day 14	14th week after discharge	15th week after discharge	Normal range
WBC	19.81	10.63	19.07	15.8	10.38	6.2	5.22	3.5–9.5 × 10^9^/L
RBC	3.34	2.6	2.6	2.6	2.5	4.6	4.3	3.8–5.1 × 10^12^/L
HGB	94	73	73	72	71	131	121	115–150 g/L
PLT	103	59	68	81	157	260	245	125–350 × 10^9^/L
NEUT%	88.9	76	84	81	75	56	57	40%–75%
LYMPH%	4.6	13	8	10	15	36.1	33	20%–50%
EO%	0	0	0.1	0.3	0.7	1.6	1.9	0.4%–8%

**Table 2:** Dynamic changes of liver and kidney function in our hospital.

	Hospitalization day 2	Hospitalization day 3	Hospitalization day 4	Hospitalization day 5	Hospitalization day 14	14th week after discharge	15th week after discharge	Normal range
TP	58.6	46.5	47	53.6	64.3	75.41	73.3	65–85 g/L
ALB	38.1	28.7	29.5	32.7	36.1	43.84	42.7	40–55 g/L
GLB	20.5	17.8	17.5	20.9	28.2	31.57	30.6	20–40 g/L
ALT	888.6	460.1	342.7	232.5	66.7	18.27	22.1	0–40 U/L
AST	131.4	74.1	68.9	54.1	32.2	31.44	30.6	0–35 U/L
ALT/AST	6.76	6.21	4.97	4.3	2.07	0.58	0.72	0.5–2
TBIL	33.7	30.6	25.1	24.5	26.9	13.36	17.3	0–21 µmol/L
DBIL	22.8	15.4	13.9	13.5	12.5	3.8	12.3	0–20 µmol/L
GR	138	91.3	88.5	90.6	91.7	54.6	40.6	33–73 U/L
UREA	34.04	13.49	17.17	10.88	8.08	7.19	6.8	2.86–8.2 mmol/L
CREA	837.8	340	497.1	372.7	438	69.62	69.2	44–106 mmol/L

On the 1st day of admission, the patient developed chest tightness and dyspnea, accompanied by delirium. Considering her previous use of cough medication and the admission workup findings, the preliminary diagnoses were acute liver injury, acute kidney injury (AKI), and severe pneumonia, potentially drug-related. She was treated with oxygen inhalation; anti-infective therapy (cefotaxime sodium 3 g, 3 times a day); cough suppression and bronchodilation (inhalation of fluticasone propionate 1 mg, acetylcysteine 3 mL, and trypsin 25,000 U, 3 times daily); hepatoprotective therapy (magnesium isoglycyrrhizinate 200 mg once daily and ornithine aspartate 5 g once daily); acid suppression and gastric protection (pantoprazole sodium 80 mg, twice daily); as well as bedside continuous renal replacement therapy. The recorded 24-hour urine outputs were 10 mL on day 1, 60 mL on day 2, and 150 mL on day 3. After 3 days of treatment, her condition stabilized, and she was transferred to our department for continued care. On the day of transfer, her 24-hour urine output was 35 mL, indicating persistent anuria. At this point, the patient strongly requested removal of the urinary catheter; in consideration of her wishes and the elevated risk of urinary tract infection associated with prolonged catheterization, the catheter was removed, and both she and her family were instructed to monitor her daily urine output. On the 5th day post-admission, the patient again experienced chest tightness and dyspnea, along with restlessness, cyanosis of the lips, multiple cutaneous ecchymoses, diffuse wet rales in both lungs, and pronounced systemic edema. Acute left-sided heart failure was suspected, and she immediately received high-flow oxygen therapy, sedation (dexmedetomidine hydrochloride 0.04 mg/h by intravenous infusion), cardiac support (deslanoside 1.2 mg/h by intravenous infusion), vasodilator therapy (nitroglycerin 2 mg/h by intravenous infusion), continuous renal replacement therapy, and further supportive measures. During this time, her daily urine output remained below 100 mL. To determine the cause of her anuria, the Self-Inquiry Murtagh safe diagnosis and treatment^[3]^ was employed, which involves the following considerations: (1) What are the common diseases that present with these symptoms or signs? (2) What significant conditions must not be overlooked? (3) Are there any etiologies that may be easily missed? (4) Could there be a concealed underlying disease? (5) Is there anything the patient has not yet disclosed?

Based on the above clinical reasoning, further consideration revealed that the patient’s acute abdominal pain and diarrhea upon admission could stem from various etiologies, such as acute inflammatory infections, drug or biological poisoning, gastrointestinal ulcers, or tumors. Laboratory results (complete blood count and inflammatory markers) suggested infection, but stool analysis, stool culture, fecal occult blood test, blood culture, and tumor markers were all normal, leaving the possibility of acute inflammation or acute poisoning open. Renal dysfunction commonly occurs in chronic kidney disease or AKI. However, given this patient’s lack of prior hypertension or anemia and normal renal ultrasound findings, chronic kidney disease could be preliminarily ruled out. According to the latest Chinese clinical practice guidelines for AKI, the initial diagnosis relies on abnormal laboratory tests, particularly an absolute or relative increase in serum creatinine.^[4]^ As the patient had 1 day of anuria and a serum creatinine level of 837.8 μmol/L upon admission, a diagnosis of AKI was established. AKI can be categorized into prerenal, intrinsic renal, and postrenal causes. Subsequent comprehensive testing for autoimmunity, anti-glomerular basement membrane antibodies, and ANCA showed no abnormalities. Additional information obtained from the previous hospital indicated an ALT of 5793 U/L and AST of 9249 U/L—approximately 145-fold and 265-fold above the normal range, respectively—pointing to acute liver failure, possibly acute hepatitis or acute poisoning. However, the patient’s hepatitis panels were normal. After further investigations and a detailed history of her medication usage and dietary intake, it was ultimately discovered that she had ingested the gallbladder of a black carp weighing about 4 kg prior to the onset of her symptoms. The final diagnosis was fish gallbladder poisoning and MODS.

On the 8th day of admission, the patient’s urine output began to increase but remained in the oliguria range, at which point standard hemodialysis was initiated. On the 12th day, both the patient and her family believed her overall condition had improved and requested discharge. However, repeat assessments indicated that her renal function had not fully recovered, her urine output remained low, and she still required maintenance hemodialysis with ongoing observation of renal function and urine volume. According to the Chinese Expert Consensus on Vascular Access for Hemodialysis, a temporary jugular catheter should not be used for more than 4 weeks in principle; if placement is anticipated for 4 weeks or longer, a long-term jugular dialysis catheter is recommended. Given the patient’s current urine output and renal status, and with her and her family’s informed consent, a long-term jugular catheter was placed under local anesthesia on the following day. She was advised to continue regular hemodialysis (2–3 times per week) following discharge, monitor her urine output, and undergo weekly renal function tests. Additionally, she was instructed to take Jianpishengxue tablets (containing *Codonopsis pilosula*, *Poria cocos*, *Astragalus membranaceus*, etc, traditionally believed to strengthen the spleen, promote digestion, and nourish the blood) at 1.8 g, 3 times daily, and Hugan granules (comprising *Bupleurum chinense*, *Artemisia capillaris*, *Isatis indigotica*, etc, which may protect the liver and lower transaminase levels) at 1.5 g, 3 times daily. Follow-up phone calls revealed that the patient continued regular dialysis at a local hospital, during which her urine output gradually increased and her renal function steadily improved on repeat tests. Three months later, follow-up examinations at our hospital showed normalization of blood counts and liver and kidney function, after which the dialysis catheter was removed in our department, as shown in Tables 1 and 2.

## 3. Discussion

The traditional Chinese medical classic “Compendium of Materia Medica” believes that fish gall has the effects of clearing away heat and detoxifying, and improving eyesight. There are also folk prescriptions indicating that ingesting fish gall may be beneficial for sore throat, high blood pressure, bronchitis, asthma, itchy skin, and treating eye diseases. Therefore, people have the habit of swallowing raw fish gall, especially those living in rural areas, who are prone to fish gall poisoning.^[5,6]^ The patient in this case is a middle-aged female farmer who lives in a rural area. She has a low level of education and limited medical knowledge. The patient believed in local folk remedies that fish gall is a safe food with medicinal value, and lacked awareness of the dangers of fish gall bladder toxicity, so she accidentally ate fish gall bladder. When the first doctor saw her, she did not tell him about her history of taking fish gall bladder, and denied eating unclean or toxic food before the onset of the disease. In addition, the patient was transferred to the Department of Critical Care Medicine of our hospital from another hospital, and then transferred to our department, resulting in our doctors not being exposed to first-hand clinical data, and the diagnosis and treatment of the patient were not detailed enough, and the medical history was not complete. Based on the trust in the initial diagnosis of the first doctor, clinical thinking anchoring occurred, which led to a delay in the diagnosis of the cause of fish gall bladder poisoning. Subsequently, through the murtagh safe diagnosis and treatment method, the patient’s medical history was repeatedly questioned and repeatedly thought back, and the cause of the disease was finally diagnosed.

Fish gall poisoning is caused by the ingestion of fish gall containing bile toxins, which is common in *Mylopharyngodon piceus*, grass carp, etc. The main components of the toxins include bile salts, histamine, hydrocyanic acid, sodium cyprinid sulfate, etc, which can damage the liver, kidneys, brain cells and myocardial cells. Histamine and hydrocyanic acid will inhibit cytochrome oxidase, block cellular energy metabolism, and lead to necrosis of proximal renal tubular epithelial cells, while sodium cyprinid sulfate will cause direct toxic damage to lysosomes, leading to multiple organ dysfunction.^[7–9]^ Gastrointestinal symptoms appear first, usually 5 to 12 hours after ingestion of fish gallbladders, while AKI appears around 3 to 6 days later and is the most common and most serious consequence, accounting for 91.7% of the causes of death.^[10]^ There have been reports of fish gallbladder poisoning leading to gastrointestinal diseases and AKI both at home and abroad. In a few cases, clinical manifestations such as sepsis, myocarditis, and hypoplasia of the 3 blood systems have also been reported.^[8,11]^ In addition, some foreign scholars have found that eating cooked fish gallbladders can also lead to fish gallbladder poisoning, and the degree of poisoning is no different from that of raw fish gallbladders.^[12,13]^ Previous studies have reported the presence of 5α-cyprinol sulfate in the bile of grass carp, a bile salt with significant hepatotoxic and nephrotoxic effects that can lead to acute tubular necrosis and hepatocellular injury.^[14–16]^ For instance, in cases reported by Lim et al,^[14]^ patients exhibited initial gastrointestinal symptoms followed by a rapid progression to acute renal failure. Liver injury in these cases manifested as a moderate elevation in liver enzymes, which resolved relatively quickly. Similarly, Chan et al^[15]^ found that acute kidney injury typically occurred 48 to 72 hours after the consumption of fish bile, while liver dysfunction usually subsided on its own within a few days, though renal function took longer to recover. According to Kung et al,^[17]^ fish bile toxins may induce cell death by inhibiting cytochrome oxidase, promoting calcium influx, and damaging the stability of lysosomal membranes. Furthermore, Rudiansyah et al^[16]^ and Haque et al^[18]^ emphasized that, apart from causing hepatic and renal impairment, these toxins might also trigger severe MODS, presenting as serious electrolyte disturbances and circulatory system injury, underscoring the need for early hemodialysis and supportive therapy. In a recent series of cases reported by Mohanty,^[19]^ fish bile toxins were again shown to precipitate acute renal failure, with concomitant severe liver injury and neurological symptoms, suggesting that their toxicity extends to multiple organ systems.

In the present case, the patient’s clinical manifestations were largely consistent with those described in previous literature, such as marked early gastrointestinal symptoms followed by a rapid and severe deterioration in hepatic and renal function. However, unlike the majority of reported cases, the patient in this case presented with abdominal pain and diarrhea as the first symptoms, accompanied by a progressive decrease in hemoglobin. The possibility of acute upper gastrointestinal bleeding caused by fish gall poisoning cannot be ruled out. Acute liver damage may occur about 1 day after taking fish gall, with ALT and AST reaching as high as 5793 and 9249 U/L, respectively. Renal function began to progressively worsen about 1 day after taking fish gall, and could reach as high as 837.8 mmol/L in about 1 week, accompanied by anuria. In addition, the patient also had corresponding symptoms such as acute left heart failure, lung infection, polyserostomy and nervous system. These features are rarely reported in the existing literature, suggesting that the clinical presentation in this case is both more complex and more severe, and thus warrants special attention in clinical practice. It can be seen that fish gall poisoning is a serious health problem that can endanger life at any time, and should be given more attention and vigilance.

The treatment principle of fish gallbladder poisoning is early detection, early diagnosis, and early treatment. Currently, there is no effective antidote. Comprehensive treatment is used, focusing on the prevention and treatment of AKI and taking multiple organs into consideration.^[6,20]^ After confirming the history of fish bile poisoning, the first thing to do is to use emetics, gastric lavage, catharsis, activated carbon adsorption and other methods to expel the toxins that have not been absorbed by the body. At the same time, blood purification should be performed as soon as possible (hemodialysis, filtration, adsorption and (or) plasma exchange can be used as appropriate) to minimize the blood concentration of fish bile toxins. On this basis, the changes in the functional indicators of tissues and organs such as the gastrointestinal tract, liver, kidney, pancreas, blood, cardiovascular and nervous systems should be closely monitored, and reasonable organ protection and supportive symptomatic treatment should be given in a timely manner, as well as targeted psychological care based on standardized care.^[10,21]^ In this case, the patient adopted blood purification and corresponding symptomatic support as soon as possible after developing MODS, which was very helpful for his good outcome. In addition, it is worth noting that the jugular vein temporary dialysis catheter is usually used for hemodialysis treatment of AKI patients, but the patient in this case needed to maintain dialysis treatment due to her condition, so she was replaced with a jugular vein long-term dialysis catheter later. Therefore, for patients with fish gallbladder poisoning who need hemodialysis treatment, in order to avoid the patient suffering from invasive catheter implantation for the second time, we may consider implanting a jugular vein long-term dialysis catheter as the first choice.

## 4. Conclusion

In summary, there are still many people who take fish gall and suffer from fish gall poisoning. Clinical physicians and health departments should increase the publicity and education of fish gall poisoning knowledge, actively strengthen medical science education, especially in rural areas, to improve people’s awareness and vigilance, and do not easily believe in folk remedies, prevent fish gall poisoning, and reduce the medical and economic burden on families and society. In addition, in clinical work, patients should be interviewed comprehensively, and detailed drug history, food history, symptoms, signs, laboratory, and imaging examination results should be traced. The diagnosis of patients transferred from other hospitals or other departments should not be easily accepted. Careful clinical thinking and dynamic differential diagnosis ideas should be maintained at all times, and adjusted as the situation changes. When the diagnosis is unclear, some techniques can be used to assist, such as murtagh safe diagnosis and treatment method.

## Author contributions

**Conceptualization:** Xiang Chen, Jing-Ping Cheng, Ling-Ling Xu, Ying-Chun Zhang.

**Data curation:** Xiang Chen, Jing-Ping Cheng, Ying-Chun Zhang.

**Formal analysis:** Xiang Chen, Ying-Chun Zhang.

**Investigation:** Xiang Chen, Jing-Ping Cheng, Ling-Ling Xu, Jia-Xin Chen, Ying-Chun Zhang.

**Methodology:** Xiang Chen.

**Project administration:** Xiang Chen.

**Resources:** Xiang Chen, Jing-Ping Cheng.

**Validation:** Jing-Ping Cheng, Ling-Ling Xu.

**Visualization:** Xiang Chen, Jing-Ping Cheng, Jia-Xin Chen.

**Writing – original draft:** Xiang Chen, Jing-Ping Cheng, Ling-Ling Xu, Ying-Chun Zhang.

**Writing – review & editing:** Xiang Chen, Jing-Ping Cheng, Ling-Ling Xu, Jia-Xin Chen, Ying-Chun Zhang.

## References

[1] Wu HL Chen YH Zhuang LH. Study on cholestrotoxin fishes in China. J Shanghai Fisheries Univ. 2001;10:102–8.

[2] Li X. Diagnosis and treatment of 2 patients with acute fish bile poisoning. Chin Commun Physician. 2022;38:39–41.

[3] Liu XH Wang LL Zhai H. Attach importance to the clinical thinking of general practitioners and improve the diagnosis and treatment level of general practitioners: experience in the diagnosis and treatment of RS3PE syndrome. J Clin Rational Drug Use. 2022;15:173–5.

[4] National Clinical Research Center for Chronic Kidney Disease, Chinese Nephrologist Association, Expert Group on AKI Guidelines. Chinese clinical practice guideline for acute kidney injury. National Med J China. 2023;103:3332–66. 10.3760/cma.j.cn112137-20230802-00133 37963734

[5] Yan W Wang Z Lu S. Analysis of factors related to prognosis and death of fish bile poisoning in China: a retrospective study. Basic Clin Pharmacol Toxicol. 2020;127:419–28. 32441465 10.1111/bcpt.13447

[6] Zhang L Zhang WC. Research progress in the clinical diagnosis and treatment of fish bile poisoning. Shandong Med. 2010;50:113–4.

[7] Sun CC Kan TZ Yu GC. Multiple organ dysfunction syndrome caused by acute fish bile poisoning: a case report. Chin J Occup Health Dis. 2022;40:700–2. 10.3760/cma.j.cn121094-20210520-00260 36229219

[8] Mahakur AC Mahapatra HS. Acute kidney injury following ingestion of raw fish gallbladder of Indian crap (Labeo Rohita): thirty case series during 1975-2018. Indian J Nephrol. 2023;33:35–9. 37197044 10.4103/ijn.ijn_555_21 PMC10185011

[9] Asakawa M Noguchi T. Food poisonings by ingestion of cyprinid fish. Toxins. 2014;6:539–55. 24476713 10.3390/toxins6020539 PMC3942750

[10] Feng Z Wen H. Prevention and treatment of biotoxin kidney injury. Chin J Liberation Army Med. 2019;44:550–4.

[11] Zhou LN Dong SS Zhang SZ Huang W. Renal failure and hepatitis following ingestion of raw grass carp gallbladder: a case report. World J Clin Cases. 2021;9:943–50. 33585643 10.12998/wjcc.v9.i4.943 PMC7852650

[12] Singh SR Sahu SS Vasudevan G Thamizhraj M Mohanty MK. Acute renal failure and hepatopathy consequent upon ingestion of cooked gallbladder of Indian carp: a case report. J Family Med Primary Care. 2024;13:381–3. 38482284 10.4103/jfmpc.jfmpc_1388_23 PMC10931867

[13] Sovann K. Acute kidney injury due to fish gallbladder ingestion: a case report from Cambodia. Blood Purif. 2017;44(Suppl 1):22–5. 28869954 10.1159/000479579

[14] Lim PS Lin JL Hu SA Huang CC. Acute renal failure due to ingestion of the gallbladder of grass carp: report of 3 cases with review of literature. Ren Fail. 1993;15:639–44. 8290711 10.3109/08860229309069416

[15] Chan DW Yeung CK Chan MK. Acute renal failure after eating raw fish gall bladder. British Med J (Clinical Research Ed.). 1985;290:897. 10.1136/bmj.290.6472.897 PMC1417670 3919834

[16] Rudiansyah M Lubis L Bandiara R. Java barb fish gallbladder-induced acute kidney injury and ischemic acute hepatic failure. Kidney Int Reports. 2020;5:751–3. 10.1016/j.ekir.2020.03.014 PMC7210697 32405599

[17] Kung SW Chan YC Tse ML Lau FL Chau TL Tam MKP. Acute renal failure and hepatitis following ingestion of carp gallbladder. Clin Toxicol (Philadelphia, Pa.). 2008;46:753–7. 10.1080/15563650701687450 19238734

[18] Haque MR Ahmed SM Chowdhury TA Rahim MA Ananna MA Iqbal S. Concurrent acute kidney injury and acute hepatitis resulting from raw fish gallbladder ingestion: a preventable cause of community acquired organ damage. Birdem Med J. 2021;11:70–1.

[19] Mohanty NR. Fish gallbladder poisoning presenting with acute renal failure and hepatitis: myth or a fact: a case series. Indian J Critical Care Med. 2023;27:1–3.36756479

[20] Fu XJ Han XQ Zhou MY. Emergency nursing care of a patient with acute herring bile poisoning with left kidney hemorrhage. J PLA Nurs. 2013;30:46–7.

[21] Wang WY Chen Y Tong SR. Rescue experience of multiple organ injury mainly caused by severe liver injury caused by grass carp bile poisoning. Liver. 2022;27:106–8.

